# Supplementary material for: Sleep disorders and risk of infertility: A meta-analysis of observational studies
Source: PLoS One. 2023 Oct 31;18(10):e0293559. doi: 10.1371/journal.pone.0293559 (PMC10617707; doi:10.1371/journal.pone.0293559)
Supplement: S1 Table — (DOCX) [file pone.0293559.s002.docx]

**Surporting Information**

**S1 Table.** The NOS scale and AHRQ scale for studies

NOS Scale：

| Author | Year | NOS Quality Assessment Scale (Cohort Study)  Selection Comparability Outcome All | | | | Source of case simple | Number of case |
| --- | --- | --- | --- | --- | --- | --- | --- |
| I-Duo Wang | 2018 | ⚝ ⚝ ⚝ ⚝ | ⚝ ⚝ | ⚝ ⚝ ⚝ | 9 | Taiwan National Health Insurance Research Database (NHIRD) | 50,154 |
| Lauren Anne Wise | 2019 | ⚝ ⚝ ⚝ ⚝ | ⚝ | ⚝ ⚝ ⚝ | 8 | North American couples enrolled during the preconception period (2013–2017) | 1176 |
| Sydney Kaye Willis | 2019 | ⚝ ⚝ ⚝ ⚝ | ⚝ | ⚝ ⚝ ⚝ | 8 | Women aged 21-45 years living in Canada or the US | 6873 |

| Author | Year | NOS Quality Assessment Scale (Case-Control Study)  Selection Comparability Exposure All | | | | Source of case simple | Number of case |
| --- | --- | --- | --- | --- | --- | --- | --- |
| Pin-Yao Lin | 2022 | ⚝ ⚝ ⚝ | ⚝ ⚝ | ⚝ ⚝ | 7 | the Longitudinal Health Insurance Database 2000 (LHID 2000) spanning the period 1997–2013 | 63,705 |
| Zhu Wei Lim | 2021 | ⚝ ⚝ ⚝ | ⚝ ⚝ | ⚝ ⚝ | 7 | National Health Insurance program | 7200 |
| Yi-Han Jhuang, | 2021 | ⚝ ⚝ | ⚝ ⚝ | ⚝ ⚝ | 6 | the Longitudinal Health Insurance Database, a subset of the NHIRD | 23,035 |

AHRQ Scale

| Studies | Define the source | List inclu-sion/exclu-sion criteria | Indicate time period used for identifying patients | Whether subjects were consecutive | Whether subjective components were masked | Assessments for quality assurance | Explain exclusions from analysis | Assess or control confound | Explain how missing data were handled | Summarize response rates and completeness of data | Clarify follow-up data | Total (0-11) |
| --- | --- | --- | --- | --- | --- | --- | --- | --- | --- | --- | --- | --- |
| Zhu Liang  2022 | 1 | 1 | 1 | Not sure | 1 | 0 | 1 | 0 | 0 | 1 | 0 | 6 |
| Jinyan Zhao  2023 | 1 | 1 | 1 | Not sure | 1 | 1 | 1 | 1 | 0 | 0 | 0 | 7 |
