# Supplementary material for: Sleep disorders and risk of infertility: A meta-analysis of observational studies
Source: PLoS One. 2023 Oct 31;18(10):e0293559. doi: 10.1371/journal.pone.0293559 (PMC10617707; doi:10.1371/journal.pone.0293559)
Supplement: S2 Table — (DOCX) [file pone.0293559.s003.docx]

**Surporting Information**

**S2 Table.** Search Strategy

Pubmed (65)

| **Search** | **Query** | **Items found** |
| --- | --- | --- |
| #1 | "infertility"[MeSH Terms] | 72,822 |
| #2 | "sterility"[Title/Abstract] | 19,103 |
| #3 | "reproductive sterility"[Title/Abstract] | 32 |
| #4 | "subfertility"[Title/Abstract] | 4,341 |
| #5 | "infertility"[MeSH Terms] OR "sterility"[Title/Abstract] OR "reproductive sterility"[Title/Abstract] OR "subfertility"[Title/Abstract] | 85,746 |
| #6 | "sleep disorders"[MeSH Terms] | 106,682 |
| #7 | "dyssomnias"[Title/Abstract] | 65 |
| #8 | "sleep disorders extrinsic"[Title/Abstract] | 1 |
| #9 | "limit setting sleep disorder"[Title/Abstract] | 3 |
| #10 | "nocturnal eating drinking syndrome"[Title/Abstract] | 4 |
| #11 | "adjustment sleep disorder"[Title/Abstract] | 7 |
| #12 | environmental sleep disorders[Title/Abstract] | 1,101 |
| #13 | "obstructive sleep apnea"[Title/Abstract] | 29,270 |
| #14 | "sleep deficit"[Title/Abstract] | 160 |
| #15 | "sleep disturbance"[Title/Abstract] | 11,062 |
| #16 | "chronobiology disorders"[Title/Abstract] | 17 |
| #17 | "sleep deprivation"[Title/Abstract] | 9,579 |
| #18 | "jet lag syndrome"[Title/Abstract] | 48 |
| #19 | "Parasomnias"[Title/Abstract] | 1,007 |
| #20 | "sleep wake disorders"[MeSH Terms] OR "Dyssomnias"[Title/Abstract] OR "sleep disorders extrinsic"[Title/Abstract] OR "limit setting sleep disorder"[Title/Abstract] OR "nocturnal eating drinking syndrome"[Title/Abstract] OR "adjustment sleep disorder"[Title/Abstract] OR (("environment"[MeSH Terms] OR "environment"[All Fields] OR "environmental"[All Fields] OR "environmentally"[All Fields] OR "environmentals"[All Fields]) AND "sleep disorders"[Title/Abstract]) OR "obstructive sleep apnea"[Title/Abstract] OR "sleep deficit"[Title/Abstract] OR "sleep disturbance"[Title/Abstract] OR "chronobiology disorders"[Title/Abstract] OR "sleep deprivation"[Title/Abstract] OR "jet lag syndrome"[Title/Abstract] OR "Parasomnias"[Title/Abstract] | 122,180 |
| #21 | "sleep apnea syndromes"[MeSH Terms] | 42,313 |
| #22 | "sleep hypopnea"[Title/Abstract] | 38 |
| #23 | "sleep disordered breathing"[Title/Abstract] | 8,157 |
| #24 | "sleep apnea syndromes"[MeSH Terms] OR "sleep hypopnea"[Title/Abstract] OR "sleep disordered breathing"[Title/Abstract] | 44,505 |
| #25 | #20 OR #24 | 123,325 |
| #26 | #25 AND #5 | 65 |

Cochrane (32)

| **Search** | **Query** | **Items found** |
| --- | --- | --- |
| #1 | MeSH descriptor: [Infertility] explode all trees | 4,030 |
| #2 | (Sterility):ti,ab,kw OR ("Reproductive Sterility"):ti,ab,kw OR (Subfertility):ti,ab,kw | 10,576 |
| #3 | #1 OR #2 | 14,231 |
| #4 | MeSH descriptor: [Sleep Wake Disorders] explode all trees | 10,400 |
| #5 | (Dyssomnias):ti,ab,kw OR ("Sleep Disorders, Extrinsic"):ti,ab,kw OR ("Limit Setting Sleep Disorder"):ti,ab,kw OR ("Nocturnal Eating-Drinking Syndrome"):ti,ab,kw OR ("Adjustment Sleep Disorder"):ti,ab,kw | 312 |
| #6 | ("Environmental Sleep Disorders"):ti,ab,kw OR ("Obstructive Sleep Apnea"):ti,ab,kw OR ("Sleep Deficit"):ti,ab,kw OR ("Sleep disturbance"):ti,ab,kw OR ("Chronobiology Disorders"):ti,ab,kw | 10,530 |
| #7 | ("Sleep deprivation"):ti,ab,kw OR ("Jet Lag Syndrome"):ti,ab,kw OR ("Parasomnias"):ti,ab,kw | 2623 |
| #8 | #4 OR #5 OR #6 OR #7 | 18,301 |
| #9 | MeSH descriptor: [Sleep Apnea Syndromes] explode all trees | 3,377 |
| #10 | ("Sleep Hypopnea"):ti,ab,kw OR ("Sleep-Disordered Breathing"):ti,ab,kw | 3,334 |
| #11 | #9 OR #10 | 5,829 |
| #12 | #8 OR #11 | 19,081 |
| #13 | #3 AND #12 | 32 |

Embase (1302)

| **Search** | **Query** | **Items found** |
| --- | --- | --- |
| #1 | 'infertility'/exp | 148,807 |
| #2 | sterility:ab,ti OR 'reproductive sterility':ab,ti OR subfertility:ab,ti | 26,525 |
| #3 | #1 OR #2 | 163,347 |
| #4 | 'sleep disorder'/exp | 319,374 |
| #5 | dyssomnias:ab,ti OR 'sleep disorders, extrinsic':ab,ti OR 'limit setting sleep disorder':ab,ti OR 'nocturnal eating-drinking syndrome':ab,ti OR 'adjustment sleep disorder':ab,ti OR 'environmental sleep disorders':ab,ti OR 'obstructive sleep apnea':ab,ti OR 'sleep deficit':ab,ti OR 'sleep disturbance':ab,ti OR 'chronobiology disorders':ab,ti OR 'sleep deprivation':ab,ti OR 'jet lag syndrome':ab,ti OR parasomnias:ab,ti | 78,976 |
| #6 | #4 OR #5 | 334,177 |
| #7 | 'sleep disordered breathing'/exp | 97,346 |
| #8 | 'sleep apnea syndrome':ab,ti OR 'sleep hypopnea':ab,ti | 11,714 |
| #9 | #7 OR #8 | 97,956 |
| #10 | #6 OR #9 | 334,373 |
| #11 | #3 AND #10 | 1,302 |
